# Substitution as a Mechanism for Genetic Robustness: The Duplicated Deacetylases Hst1p and Sir2p in Saccharomyces cerevisiae

**DOI:** 10.1371/journal.pgen.0030126

**Published:** 2007-08-03

**Authors:** Meleah A Hickman, Laura N Rusche

**Affiliations:** 1 Institute for Genome Sciences and Policy, Duke University, Durham, North Carolina, United States of America; 2 University Program in Genetics and Genomics, Duke University, Durham, North Carolina, United States of America; 3 Department of Biochemistry, Duke University Medical Center, Durham, North Carolina, United States of America; National Institute of Diabetes and Digestive and Kidney Diseases, United States of America

## Abstract

How duplicate genes provide genetic robustness remains an unresolved question. We have examined the duplicated histone deacetylases Sir2p and Hst1p in Saccharomyces cerevisiae and find that these paralogs with non-overlapping functions can provide genetic robustness against null mutations through a substitution mechanism. Hst1p is an NAD^+^-dependent histone deacetylase that acts with Sum1p to repress a subset of midsporulation genes. However, *hst1*Δ mutants show much weaker derepression of target loci than *sum1*Δ mutants. We show that this modest derepression of target loci in *hst1*Δ strains occurs in part because Sir2p substitutes for Hst1p. Sir2p contributes to repression of the midsporulation genes only in the absence of Hst1p and is recruited to target promoters by a physical interaction with the Sum1 complex. Furthermore, when Sir2p associates with the Sum1 complex, the complex continues to repress in a promoter-specific manner and does not spread. Our results imply that after the duplication, *SIR2* and *HST1* subfunctionalized. The single *SIR2/HST1* gene from *Kluyveromyces lactis,* a closely related species that diverged prior to the duplication, can suppress an *hst1*Δ mutation in S. cerevisiae as well as interact with Sir4p in *S. cerevisiae.* In addition, the existence of two distinct protein interaction domains for the Sir and Sum1 complexes was revealed through the analysis of a chimeric Sir2–Hst1 molecule. Therefore, the ability of Sir2p to substitute for Hst1p probably results from a retained but reduced affinity for the Sum1 complex that is a consequence of subfunctionalization via the duplication, degeneration, and complementation mechanism. These results suggest that the evolutionary path of duplicate gene preservation may be an important indicator for the ability of duplicated genes to contribute to genetic robustness.

## Author Summary

Gene duplication is an important force in evolution, as it provides a source of new genetic material. However, the mechanisms by which duplicated genes are retained and diverge are understudied at the experimental level. We have examined a pair of duplicated histone deacetylases Hst1p and Sir2p from baker's yeast, which are important for distinct types of gene repression. In this study, we show that before the duplication the ancestral histone deacetylase had both Hst1p- and Sir2p-like functions, and after the duplication Sir2p and Hst1p subfunctionalized, giving rise to two distinct proteins with normally nonoverlapping functions. Despite having partitioned the ancestral functions after the duplication, Sir2p can substitute for Hst1p in its absence by interacting with the normal partner of Hst1p. This study suggests that the evolutionary path of duplicate gene preservation may be an important indicator for the ability of duplicated genes to substitute for one another and hence protect the organism against deleterious mutations.

## Introduction

The evolutionary role of gene duplication presents a paradox. Gene duplication provides a source of new genetic material that is free of selective constraint and can evolve novel functions [1,2], but at the same time, gene duplication provides genetic robustness against deleterious mutations through redundant function [3–5]. How duplicated genes protect against null mutations while continuing to evolve different functions is at the core of the paradox. Deletion of duplicated genes results in less severe fitness phenotypes than deletion of singleton genes [6]. It has been hypothesized that duplicate gene pairs with high sequence similarity are more likely to be functionally redundant and contribute to genetic robustness against deleterious mutations, whereas duplicate gene pairs with low sequence similarity have diverged to such an extent to no longer be able to functionally complement each other. However, there is no correlation between sequence similarity between duplicates and their contribution to genetic robustness [7]. Indeed, regardless of sequence divergence, gene duplicates arising from a whole genome duplication in S. cerevisiae are less likely than singleton genes to be essential [8]. However, it remains unclear how duplicated genes that have diverged from each other in sequence and function can provide genetic robustness against deleterious mutations. Previous genome-wide studies have been limited in their ability to deduce a molecular mechanism for gene duplication in genetic robustness because phenotypes were assessed without regard to gene function. In this study, we have investigated in detail how the nonredundant duplicated gene pair *HST1* and *SIR2* in Saccharomyces cerevisiae functions to provide genetic robustness against null mutation.

In *S. cerevisiae,* Hst1p is an NAD^+^-dependent histone deacetylase that acts with the protein Sum1p to repress a subset of midsporulation genes [9–12]*.* Hst1p deacetylates histones H3 and H4 [9], and this deacetylation is thought to be important for its repressive function. Sum1p is a DNA binding protein that associates with the middle sporulation element (MSE), a conserved sequence found primarily in midsporulation gene promoters [12–14]. The third member of the Sum1 complex, Rfm1p, is a small protein thought to serve an architectural role by associating with both Sum1p and Hst1p [10]. There are two noteworthy differences among phenotypes of *sum1*Δ, *rfm1*Δ, and *hst1*Δ null mutations. First, the subset of midsporulation genes derepressed in these backgrounds differs. One group of genes requires Rfm1p and Hst1p in addition to Sum1p; the other group requires only Sum1p for repression [10]. Second, for genes that are repressed by both Sum1p and Hst1p, there is a difference in the level of derepression of target loci between *sum1*Δ and *hst1*Δ strain backgrounds [10]. Deletion of Sum1p results in a strong derepression of target midsporulation genes, whereas deletion of Hst1p results in a modest derepression of the same target midsporulation genes. It has been unclear what contributes to this difference in phenotypes.

Hst1p is a member of the Sir2 family of NAD^+^-dependent deacetylases. These enzymes have a highly conserved catalytic core domain and variable terminal extensions. Deacetylases of the Sir2 family are present ubiquitously across life, with family members in bacterial, archael, plant, fungal, and animal species [15]. Biological functions of these family members are diverse, with roles in transcriptional silencing, chromosome stability, cell cycle progression, and aging [16]. The distinct and variable functions of Sir2 family members are a result of multiple duplication events with subsequent diversification of substrates and functions. In S. cerevisiae there are five NAD^+^-dependent deacetylases: Sir2p, the founding member of the entire family; Hst1p; Hst2p; Hst3p; and Hst4p [17–19]. Hst2p is a predominantly cytoplasmic protein [20], but may have a cell cycle-specific nuclear localization [21,22]. Hst3p and Hst4p deacetylate lysine 56 on histone H3 and are involved in cell cycle and DNA damage checkpoints that modulate chromatin, enabling replication and condensation to occur properly [17,23,24].

Of these five NAD^+^-dependent deacetylases in *S. cerevisiae, HST1* is the most closely related to *SIR2*. *HST1* and *SIR2* arose in a whole genome duplication in the ancestry of *Saccharomyces* species, which occurred approximately 100 million years ago [25–27]. Overall sequence conservation between *SIR2* and *HST1* is 63% (76% similar) [17] with three conserved regions: the well-conserved catalytic core domain with 82% sequence identity (92% similarity) and lesser-conserved regions in the N terminus and the extreme C-terminal tail [28]. Despite their sequence similarity, *HST1* and *SIR2* have non-overlapping functions [10,17,18]. We have used *SIR2* and *HST1* as a case study to understand diversification of the Sir2 family through duplication.

In contrast to the promoter-specific mechanism of transcriptional repression in which Hst1p participates, Sir2p is involved in long-range silencing. Sir2p acts with Sir3p and Sir4p to generate a special chromatin structure that silences the mating-type loci and telomeres [29]. *Cis*-acting silencer elements recruit the four Sir proteins. Then, Sir2p, Sir3p, and Sir4p spread along the chromosome [30–32]. The histone deacetylase activity of Sir2p is required for the spreading of all three Sir proteins [30,32]. Sir3p and Sir4p bind preferentially to deacetylated tails of histones H3 and H4 [33]. Sir2p deacetylates nearby nucleosomes, creating new high affinity binding sites for Sir3p and Sir4p, which in turn recruit additional Sir2p to the newly deacetylated nucleosomes. As the Sir proteins spread, they generate a specialized chromatin structure that is restrictive to transcription and independent of DNA sequence. Sir2p is also part of the RENT complex (Regulator of nucleolar silencing and telophase exit), which modulates chromatin structure in the rDNA repeats [34]. The RENT complex does not contain the other Sir proteins, and its mechanism of action is less well understood.

We examined whether the difference in derepression of midsporulation genes between *sum1*Δ and *hst1*Δ strains is a consequence of Sir2p, the closest paralog to Hst1p substituting for Hst1p in the absence of Hst1p. We have shown through genetic and biochemical means that Sir2p can substitute for Hst1p, and this phenomenon is a product of the path of evolutionary divergence after the duplication of these two genes.

## Results

### Sir2p Substitutes for Hst1p in an *hst1*Δ Background

Gene expression data indicate that deletion of *HST1* derepresses target genes modestly, compared to the level of derepression observed in a *sum1*Δ background (unpublished data) [10]. These results suggest that either deacetylation is not critical for gene repression or another deacetylase acts at these promoters in the absence of Hst1p. To identify other deacetylases that may function in the absence of Hst1p, the four other known NAD^+^-dependent deacetylases, *SIR2, HST2, HST3,* and *HST4* were deleted in combination with *HST1*. To assay levels of expression in these double deletion backgrounds, a p*_GAS2_*-*HIS3* reporter was used. The *GAS2* promoter is not strongly induced in the absence of Hst1p but is greatly induced in the absence of Sum1p [10]. In addition, the promoter contains a MSE and is reported to bind Sum1p [35]. If another deacetylase contributes to repression at this promoter in the absence of Hst1p, then deletion of both deacetylases should derepress the p*_GAS2_*-*HIS3* reporter to a greater extent than deletion of Hst1p alone. Increased expression was observed in the *hst1*Δ *sir2*Δ double deletion strain compared to the *hst1*Δ strain (Figure 1A). The other double deletions, *hst1*Δ *hst2*Δ, *hst1*Δ *hst3*Δ, and *hst1*Δ *hst4*Δ, did not display any difference in derepression compared to the single *hst1*Δ background.

**Figure 1:**
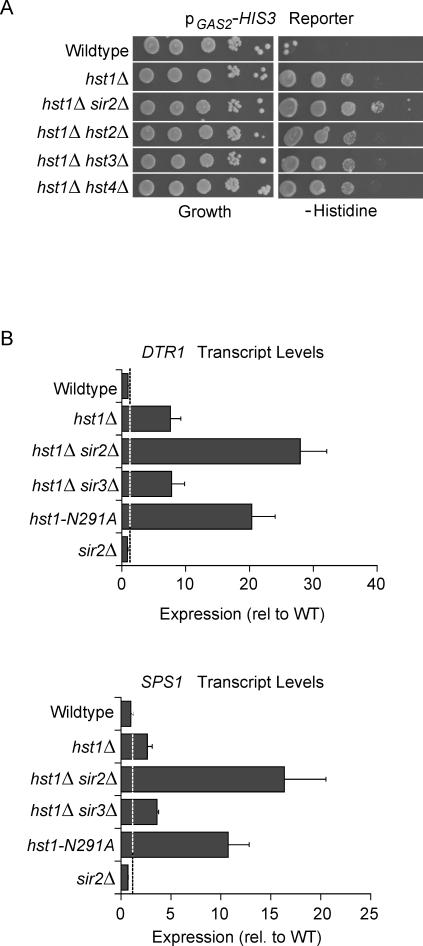
Sir2p Contributes to Repression of Sum1p-Repressed Genes in the Absence of Hst1p (A) Expression of the Sum1p-repressed p*_GAS2_-HIS3* reporter was tested in wild-type (LRY1453)*, hst1*Δ (LRY1454)*, hst1*Δ *sir2*Δ (LRY1422)*, hst1*Δ *hst2*Δ (LRY1686), *hst1*Δ *hst3*Δ (LRY1704), and *hst1*Δ *hst4*Δ (LRY1687) strain backgrounds. A ten-fold dilution series of each strain was plated on rich medium (growth) or medium lacking histidine (−histidine) and photographed after 3 d of growth at 30 °C. The most concentrated samples on rich medium and the most dilute samples on selective medium were omitted. Thus, the first spot on complete medium is equivalent to the second spot on medium lacking histidine. (B) Expression of the Sum1p-repressed genes *DTR1* and *SPS1* was assessed in wild type (W3031-a), *hst1*Δ (LRY198), *hst1*Δ *sir2*Δ (LRY333)*, hst1-N291A* (LRY1306), *sir2*Δ (LRY1079), and *hst1*Δ *sir3*Δ (LRY345) strains. RNA was extracted from logarithmically growing cells and analyzed by quantitative RT-PCR. *DTR1* and *SPS1* transcript levels were normalized to *ACT1* transcript levels and then compared to the wild-type strain to measure gene induction. A value of one (dashed line) corresponds to no induction.

To extend this observation and examine more quantitatively the difference between *hst1*Δ and *hst1*Δ *sir2*Δ derepression phenotypes, gene expression levels of *DTR1* and *SPS1,* two midsporulation genes repressed by Sum1p and Hst1p [10], were measured by quantitative reverse transcriptase (RT)-PCR in wild-type, *hst1*Δ, and *hst1*Δ *sir2*Δ strains. *DTR1* and *SPS1* were modestly induced in an *hst1*Δ background (Figure 1B) in accordance with previous observations [10]. Consistent with the results of the p*_GAS2_*-*HIS3* reporter (Figure 1A), the induction of *DTR1* and *SPS1* in an *hst1*Δ *sir2*Δ strain was dramatically greater than in an *hst1*Δ strain (Figure 1B). It should be noted that although derepression of midsporulation genes in an *hst1*Δ *sir2*Δ background was greater than was observed in an *hst1*Δ background, this derepression was not to the level observed in a *sum1*Δ strain (unpublished data). These results indicated that Sir2p contributed to the repression of midsporulation genes in the absence of Hst1p.

To determine whether the increased expression of Hst1p-repressed loci in an *hst1*Δ *sir2*Δ background resulted specifically from the loss of Sir2p or was an indirect effect due to the disruption of Sir-mediated silencing, the induction of *DTR1* and *SPS1* was examined in an *hst1*Δ *sir3*Δ background. If the observed increased expression resulted from the loss of Sir-mediated silencing, then the *hst1*Δ *sir3*Δ strain should have the same level of *DTR1* and *SPS1* induction as the *hst1*Δ *sir2*Δ strain. On the other hand, if the increased gene expression observed in the *hst1*Δ *sir2*Δ strain resulted specifically from the loss of Sir2p, then retaining Sir2p while disrupting Sir-mediated silencing should resemble the *hst1*Δ phenotype rather than the *hst1*Δ *sir2*Δ phenotype. The level of *DTR1* and *SPS1* induction in the *hst1*Δ *sir3*Δ strain was comparable to the *hst1*Δ strain and dramatically less than the *hst1*Δ *sir2*Δ strain (Figure 1B)*.* We conclude that it was the absence of the Sir2p deacetylase and not disruption of Sir-mediated silencing that contributed to the elevated level of *DTR1* and *SPS1* gene expression in the *hst1*Δ *sir2*Δ background.

It is possible that Sir2p always contributes to the repression of the midsporulation genes. Alternatively, the absence of Hst1p could provide an opportunity for Sir2p to associate with the Sum1 complex, such that Sir2p only contributes to this repression in the absence of Hst1p. To test the latter hypothesis, we characterized *DTR1* and *SPS1* expression in a strain in which Hst1p was enzymatically inactive, such that the mutant Hst1p could not contribute to deacetylation yet was present and could physically block Sir2p from acting in its place. To inactivate Hst1p, a single amino acid substitution, N291A (described in [36]), was used, analogous to a characterized substitution in Sir2p (N345A), which has been shown to be enzymatically inactive [37] but structurally intact [38]. This point mutation in Hst1p reduced deacetylation in vivo (as discussed in a subsequent subsection of the results). The *hst1-N291A* strain displayed significantly greater induction of *DTR1* and *SPS1* compared to the *hst1*Δ strain (Figure 1B). This observation suggests that Sir2p may be acting in the absence of Hst1p, but not when the mutated Hst1-N291Ap is present. Furthermore, the increased induction in the *hst1-N291A* strain compared to the *hst1*Δ strain indicates that the main function of Hst1p in repression is deacetylation.

To examine whether Sir2p normally contributes to repression of *DTR1* and *SPS1,* the expression profiles of *DTR1* and *SPS1* in a *sir2*Δ background were analyzed. If Sir2p has no role in Hst1p-mediated repression when Hst1p is present, then deleting *SIR2* alone should have no discernable phenotype compared to wild-type yeast, and repression of *DTR1* and *SPS1* should be maintained. Repression of *DTR1* and *SPS1* was maintained in a *sir2*Δ background (Figure 1B), and the *p_GAS2_*-*HIS3* reporter also remained repressed in a *sir2*Δ strain (unpublished data). These results suggest that Sir2p does not normally play a role in Sum1p-mediated repression when Hst1p is present.

### Sir2p Associates with the Sum1 Complex

To further test the hypothesis that Sir2p substitutes for Hst1p but does not normally act with Sum1p, the association of Sir2p with repressed promoters in the presence and absence of Hst1p was examined. If the substitution model is correct, Sir2p should not be enriched at repressed promoters when Hst1p is present (in wild-type or *hst1-N291A* strains) but should be recruited to these promoters in an *hst1*Δ background. Chromatin immunoprecipitation (ChIP) was used to detect HA-Sir2p or Hst1p-HA at the *DTR1* promoter in wild-type and *hst1*Δ strains. In a wild-type background, there was a high level of Hst1p-HA enrichment but no detectable enrichment of HA-Sir2p at the promoter of *DTR1* (Figure 2A). There was also no enrichment of HA-Sir2p observed in the *hst1-N291A* background (Figure 2A). These results are consistent with the model that Sir2p is absent from these promoters when Hst1p is present. However, when Hst1p was absent, there was a modest enrichment of HA-Sir2p at the *DTR1* promoter (Figure 2A). The enrichment of Sir2p was not as robust as wild-type Hst1p at these loci, suggestive of a weaker interaction between Sir2p and the Sum1 complex.

**Figure 2:**
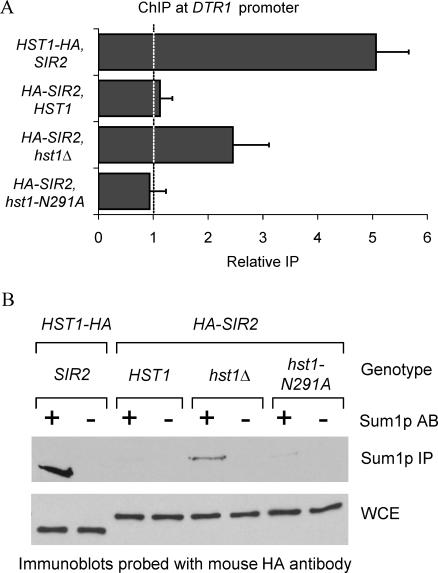
Sir2p Associates with the Sum1 Complex. (A) Association of HA-Sir2p (pRO298) with the *DTR1* promoter in wild-type (W3031-a), *hst1-N291A* (LRY1306), and *hst1*Δ (LRY198) strain backgrounds was assessed by ChIP followed by quantitative PCR. Also shown is the association of Hst1p-HA (LRY558) with the same promoters in a wild-type background. Association at the *DTR1* (B primers) promoter was normalized to an internal control, the *ATS1* promoter, which is not regulated by Hst1p or Sir2p. The y-axis represents the enrichment of *DTR1* relative to *ATS1*. A value of one (dashed line) corresponds to no enrichment. (B) The association of HA-Sir2p or Hst1p-HA with Sum1p was assessed by co-precipitation. Sum1p was immunoprecipitated from whole-cell extracts from the same strains used in (A), and the precipitated material was examined by immunoblotting with an α-mouse HA antibody to detect Hst1p-HA or HA-Sir2p.

To examine directly whether the recruitment of Sir2p to repressed promoters is due to an interaction with the Sum1 complex, co-immunoprecipitation experiments between Sum1p and HA-Sir2p in an *hst1*Δ background were performed. Hst1p and Sum1p are part of a stable complex that coprecipitates (Figure 2B) [10,11,39]. If Sir2p substitutes for Hst1p via a similar interaction with the Sum1 complex, then a physical association between these two proteins should be detectable. Sum1p was immunoprecipitated, and the immunoprecipitation samples were probed for HA-Sir2p by immunoblotting. Consistent with the substitution model, Sir2p associated with Sum1p in the *hst1*Δ background (Figure 2B). This coprecipitation of Sir2p with Sum1p was weaker than the precipitation observed for Hst1p from an equivalent amount of cell extract. This qualitative comparison is consistent with the Hst1p–Sum1p interaction being more robust than the Sir2p–Sum1p interaction and in accordance with the reduced enrichment of Sir2p compared to Hst1p observed at the promoter of *DTR1* (Figure 2A).

To test the hypothesis that the presence of Hst1p physically blocks the association of Sir2p with the Sum1 complex, the Sir2p–Sum1p interactions in wild-type and *hst1-N291A* backgrounds were examined. In the presence of Hst1p, Sir2p would not be expected to interact with the Sum1 complex, and indeed Sir2p was not observed to coprecipitate with Sum1p in wild-type yeast. There was a faint band in the *hst1-N291A* background that could be indicative of Sir2p interaction with the Sum1 complex, however this band was considerably less robust than that observed in the *hst1*Δ strain (Figure 2B). Therefore, we conclude that Sir2p is recruited to Hst1p-repressed loci through an interaction with the Sum1 complex and this recruitment only occurs in the absence of Hst1p. Presumably, Hst1p outcompetes Sir2p for association with the Sum1 complex because Hst1p has a higher affinity for the Sum1 complex.

The results in the previous section suggested that Sir2p can substitute for Hst1p but does not normally act with Sum1p. Gene expression data (Figure 1), in addition to the physical interactions described above (Figure 2), do not support the hypothesis that Sir2p plays a role in Sum1p-mediated repression when Hst1p is present. Instead, these results support the hypothesis that Sir2p and Hst1p have nonoverlapping functions in wild-type backgrounds [10,17,18].

### Sir2p Acts as a Histone Deacetylase at Sum1p-Repressed Loci

To investigate whether Sir2p acts as a deacetylase at Sum1p-repressed promoters, ChIP experiments were performed with two different histone H4 antibodies, one specific for acetylated lysine 8 (K8) and the other specific for acetylated lysine 16 (K16). The changes in acetylation of K8 or K16 at the *DTR1* promoter in *hst1*Δ, *hst1-N291A*, *hst1*Δ *sir2*Δ, and *sir2*Δ strains relative to a wild-type strain were analyzed. Loss of deacetylation by Hst1p and Sir2p at *DTR1*, such as in *hst1-N291A* and *hst1*Δ *sir2*Δ backgrounds, should result in increased acetylation of K8 and K16. Indeed, increased levels of acetylation of both K8 and K16 were observed in both *hst1-N291A* and *hst1*Δ *sir2*Δ backgrounds (Figure 3). These results parallel the patterns observed in our gene expression profiles. The single *sir2*Δ deletion did not display elevated levels of acetylated K8 or acetylated K16 at *DTR1,* providing further support for the model that Sir2p does not normally act at Hst1-repressed loci when Hst1p is present.

**Figure 3:**
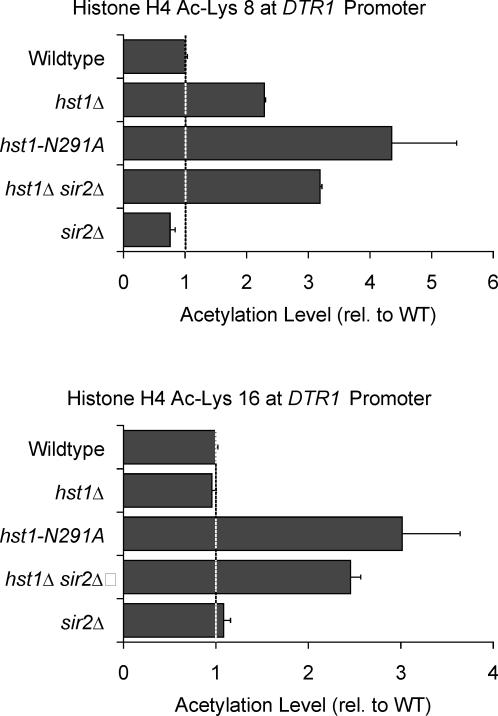
Histone H4 K8 and K16 Acetylation at the *DTR1* Promoter Increases in the Absence of Deacetylase Activity The relative levels of acetylation of H4 K8 and H4 K16 were determined in wild-type (W3031-a), *hst1*Δ (LRY198), *hst1*-*N291A* (LRY1306), *hst1*Δ *sir2*Δ (LRY333), and *sir2*Δ (LRY1079) backgrounds at the *DTR1* promoter (B primers). Histone H4 K8-Ac and K16-Ac enrichment at *DTR1* was normalized to the repressed promoter of *PHO5* as well as for total histone H4 occupancy and quantified relative to the wild-type strain.

Interestingly, when the *hst1*Δ and wild-type strains were compared, changes in acetylation were different for K8 and K16 (Figure 3). A modest increase of acetylation at K8 was observed in the *hst1*Δ background, whereas no detectable change (compared to a wild-type strain) in acetylation of K16 was noted. These results suggest that K16 is more efficiently deacetylated by Sir2p than K8 because changes in K16 acetylation were only revealed when both Hst1p and Sir2p were absent (*hst1*Δ *sir2*Δ). These data are consistent with published reports that Sir2p preferentially deacetylates H4 K16 in vitro [37,40]. Nevertheless, K16 must also be a target for deacetylation by Hst1p because K16 acetylation increased when Hst1p was nonfunctional *(hst1-N291A)* (Figure 3). In conclusion, these results indicate that Sir2p acts as a deacetylase at Sum1p-repressed promoters in the absence of Hst1p.

### Sir2p Substitution for Hst1p Is Limited by Dosage

To determine whether there is still sufficient Sir2p available to silence the mating-type loci (a primary function of Sir2p) when Sir2p is substituting for Hst1p, the ability of wild-type, *sir2*Δ, and *hst1*Δ strains to mate was assessed. If Sir2p recruitment to Sum1p-repressed loci in an *hst1*Δ background reduces the pool of available Sir2p, then silencing at the mating-type loci might be reduced, leading to diminished mating. Alternatively, if the preferred function of Sir2p is to silence the mating-type loci, then there should be no defect in mating ability in an *hst1*Δ background, even though Sir2p is substituting for Hst1p. There was no observable defect in mating ability in an *hst1*Δ background compared to a wild-type strain (Figure 4A) [17,18]. Therefore, Sir2p is more likely to silence the mating-type loci than to substitute for Hst1p. Furthermore, these results suggest that Sir2p has a higher affinity for the Sir complex than the Sum1 complex, because the ability to mate is not perturbed in the absence of Hst1p, whereas repression of midsporulation genes is not complete when Sir2p is substituting for Hst1p.

**Figure 4:**
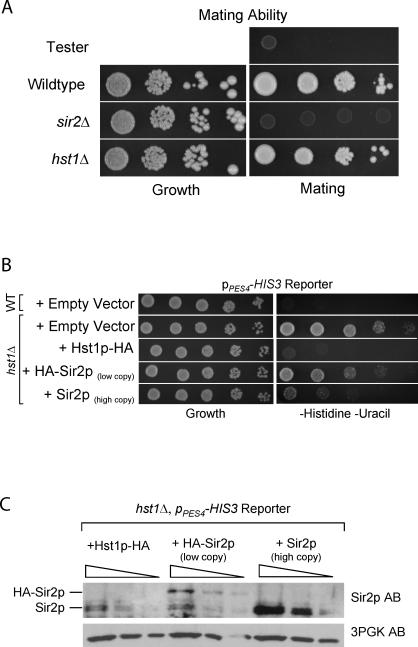
Sir2p Substitution for Hst1p Is Limited by Dosage (A) Mating ability was assayed using 10-fold serial dilutions of wild-type (W3031-a), *sir2*Δ (LRY1079), and *hst1*Δ (LRY198) strains mated against a MATα tester strain (LRY1022). Prototrophic diploids were selected on minimal plates. A 10-fold dilution series of the tester strain was plated on minimal plates as a negative control. (B) The p*_PES4_-HIS3* reporter was used to assay Sum1p-mediated repression*.* Wild-type yeast (LRY1593) were transformed with an empty vector (pRS416), and *hst1*Δ yeast (LRY1545) were transformed with an empty vector (pRS416), *HST1-HA* (pLR30), *HA-SIR2* (pRO298 [low copy]), or *SIR2* (pLP317 [high copy]). A five-fold dilution series of each strain was plated on rich medium and medium lacking histidine and uracil (to maintain plasmids) and photographed after 3 d growth at 30 °C. (C) Immunoblot analysis showing relative amounts of Sir2p protein in the strains described in part B. Endogenous Sir2p (lower band) is present in all strains. The HA-Sir2p is slightly shifted because of the epitope tag. As a loading control, 3-phosphoglycerate kinase was detected.

If the majority of Sir2p is involved in silencing the mating-type loci (and telomeres), and only a few molecules of Sir2p are available for recruitment to Hst1p-repressed loci in the absence of Hst1p, then additional copies of Sir2p may enhance repression of Hst1p-repressed loci. Overexpression of Sir2p has been reported to reduce β-Galactosidase activity from an MSE-containing promoter driving *lacZ* expression in an *hst1*Δ background [12]. To further characterize this observation, the amount of Sir2p in the cell was varied to determine whether overexpression of Sir2p enhanced its ability to substitute for Hst1p. To assay repression, a reporter construct consisting of the Sum1-repressed *PES4* promoter fused to the open reading frame of *HIS3* was utilized. In the absence of Hst1p, the *PES4* promoter is derepressed to a greater extent than the *GAS2* promoter described previously (Figure 1A), enabling an enhancement of repression to be detected. *hst1*Δ cells were transformed with low copy plasmids expressing *HST1-HA* or *HA-SIR2* and a high-copy plasmid expressing *SIR2.* The relative levels of Sir2p are shown in Figure 4C. Expression of the p*_PES4_*-*HIS3* reporter was monitored on medium lacking histidine and uracil (to ensure plasmid retention). A wild-type strain displayed no growth on selective medium, indicating that the *PES4* promoter was repressed as expected (Figure 4B). In an *hst1*Δ background, cells were able to grow on selective medium as a result of derepression of the p*_PES4_*-*HIS3* reporter (Figure 4B), demonstrating that the reporter assay is functional. Note that endogenous levels of Sir2p are present in all strains (Figure 4C). The addition of Sir2p on a low copy plasmid resulted in an enhancement of repression of p*_PES4_*-*HIS3*, and overexpression of Sir2p from a high copy plasmid enhanced repression of p*_PES4_*-*HIS3* to an even greater extent. Despite the enhancement in repression observed upon overexpression of Sir2p, repression of p*_PES4_*-*HIS3* was not complete in the absence of Hst1p. This incomplete suppression probably results from the relatively weaker interaction of the Sum1 complex with Sir2p compared to Hst1p.

It is thought that Sir2p associates directly with Sir4p but not Sir3p [41]. Therefore, additional Sir2p might become available by deleting Sir4p, which would result in a stronger repression phenotype than observed in an *hst1*Δ background. However, gene expression analysis of *DTR1* in an *hst1*Δ *sir4*Δ strain showed roughly equivalent levels of *DTR1* induction to an *hst1*Δ strain (unpublished data).

### The Sir2p–Sum1p Complex Does Not Spread

A key distinguishing feature between Hst1p and Sir2p is that Sir2p is normally part of the Sir-silencing complex that spreads along the chromosome [29], whereas the Hst1p–Sum1p complex does not spread [36]. We were interested to determine whether the Sir2p–Sum1p complex was able to spread, although the Hst1p–Sum1p complex does not spread, indicating some intrinsic property in Sir2p to promote spreading. To assess the ability of Sum1p to spread, the distribution of myc-Sum1p across the *DTR1* locus was analyzed by ChIP when the Sum1 complex was interacting with Hst1p (wild-type cells) or Sir2p (*hst1*Δ cells). There is a probable MSE sequence in the promoter of *DTR1* to which Sum1p is thought to bind (Figure 5A) [35]. When the Sum1p–Hst1p complex is present, myc-Sum1p should associate most strongly with the MSE DNA sequence and should have reduced association with the surrounding sequences (approximately 200 bp upstream and downstream of the MSE). Due to the technical limitations of shearing DNA by sonication, sequences near the binding site are also enriched in immunoprecipitated material, even if the protein does not spread. If Sir2p causes Sum1p to spread when it substitutes for Hst1p, myc-Sum1p should be more broadly distributed across the *DTR1* promoter and into the open reading frame. However, if Sir2p does not confer the ability to spread, then the distribution of myc-Sum1p across *DTR1* should not be appreciably different in *HST1* and *hst1*Δ strains. The distribution of myc-Sum1p across the *DTR1* locus remained the same regardless of which deacetylase was interacting with Sum1p (Figure 5B). Therefore, Sir2p did not cause noticeable spreading of Sum1p when substituting for Hst1p, and Sum1p continued to act as a promoter-specific repressor.

**Figure 5:**
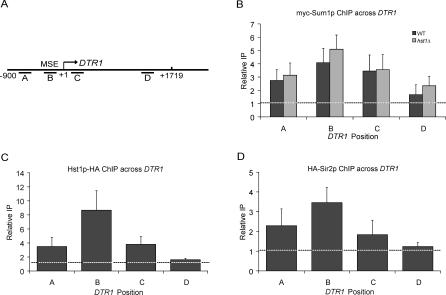
Sir2p Does Not Alter the Spreading Properties of the Sum1 Complex (A) The positions of PCR amplicons in the *DTR1* locus are shown. (B) The distribution of myc-Sum1p at the *DTR1* promoter in wild-type (LRY523) and *hst1*Δ (LRY521) strain backgrounds was assessed by ChIP followed by quantitative PCR. The y-axis represents the enrichment of *DTR1* relative to *ATS1*. A value of one (dashed line) corresponds to no enrichment. (C) The distributions of Hst1p-HA in a wild-type (LRY558) background and (D) HA-Sir2p in an *hst1*Δ (LRY198 with pRO298) background were assessed as in part B.

We extended this analysis to examine whether Sir2p itself can spread across the *DTR1* locus, even though Sum1p does not spread. The distribution of Hst1p-HA (in a wild-type background) and HA-Sir2p (in an *hst1*Δ background) across the *DTR1* locus was assessed by ChIP. As expected, Hst1p-HA had a distribution centering around the MSE and did not extend into the open reading frame (Figure 5C), indicating that Hst1p is not spreading at repressed midsporulation genes. The localization of HA-Sir2p had a similar distribution that was centered at the MSE and did not extend into the open reading frame (Figure 5D). These results demonstrate that Sir2p can act in a promoter-specific manner to repress gene expression when associated with the Sum1 complex.

### Hst1p and Sir2p Have Different Protein Interaction Domains

How do Hst1p and Sir2p maintain nonoverlapping functions when both deacetylases are present, despite considerable sequence identity and the ability of Sir2p to substitute for Hst1p? One possibility is that Hst1p and Sir2p have unique determinants that confer specificity for the Sum1 complex and the Sir complex, respectively. Because the N terminus is less conserved than the catalytic core (Figure 6A), this region may have evolved distinct specificities for either the Sir or Sum1 complex. To determine whether such determinants exist, a chimeric Sir2–Hst1p molecule was constructed in which the N terminus of Sir2p was fused to the catalytic core of Hst1p. The junction of the Sir2–Hst1 chimera was at the start of the catalytic core domain, such that amino acids 1–255 of Sir2p were fused to amino acids 201–503 from Hst1p (Figure 6A), generating HA*-*Sir2_1−255_-Hst1_201−503_p. This chimeric gene was expressed from the *SIR2* promoter. We also constructed the reverse chimera, HA-Hst1_1−200_-Sir2_256−562_p, but were not able to detect protein expression by immunoblotting (unpublished data) and continued analysis only with HA*-*Sir2_1−255_-Hst1_201−503_p.

**Figure 6:**
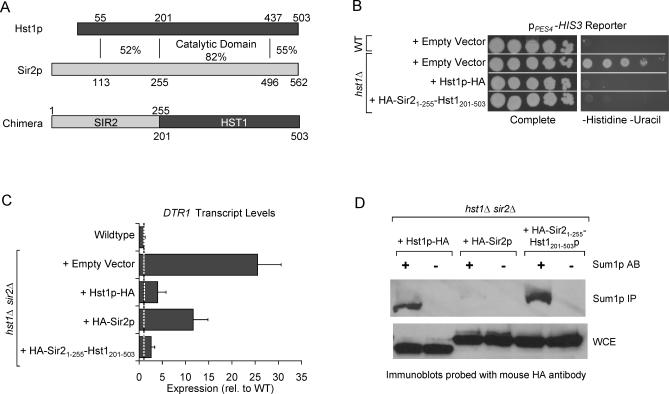
The Sir2_1−255_-Hst1_201−503_p Chimera Functions like Hst1p (A) A schematic overview of conserved domains of Sir2p and Hst1p (adapted from [28]) and the architecture of the Sir2_1−255_-Hst1_201−503_p chimera are shown. (B) The p*_PES4_-HIS3* reporter was used to assay repression by the HA-Sir2_1−255_-Hst1_201−503_p chimera (pLR488) and Hst1p-HA (pRO298) in a *hst1*Δ strain (LRY1545). A ten-fold dilution series of each strain was plated on rich medium and medium lacking histidine and uracil (to maintain plasmid) and photographed after 3 d growth at 30 °C. (C) *DTR1* gene expression was assessed by RT-PCR for *hst1*Δ *sir2*Δ (LRY333) cells transformed with an empty vector (pRS416), *HST1*-HA (pLR30), HA-*SIR2* (pRO298), or HA-Sir2_1−255_-Hst1_201−503_ (pLR488). Reported values are relative to wild-type (W303-1a) cells. (D) The association of HA-Sir2_1−255_-Hst1_201−503_p, HA-Sir2p, and Hst1p-HA with Sum1p was assessed by co-immunoprecipitation. Sum1p was immunoprecipitated from whole-cell extracts from the same strains used in (C), and the precipitated material was examined by immunoblotting with an α-mouse HA antibody to detect Hst1p-HA, HA-Sir2p, or HA-Sir2_1−255_-Hst1_201−503_p.

This chimeric protein was tested for its ability to function like Sir2p and Hst1p. If specificity for the Sir complex (in Sir2p) and specificity for the Sum1 complex (in Hst1p) is established by the N terminus, then HA*-*Sir2_1−255_-Hst1_201−503_p should only function like Sir2p. Alternatively, if specificity for the Sir or Sum1 complex is determined by the C terminus, then HA*-*Sir2_1−255_-Hst1_201−503_p should associate with the Sum1 complex and function like Hst1p. Finally, it may be that specificity for the Sir and Sum1 complex may be determined in entirely different regions of Sir2p and Hst1p. If this were true, then it may be possible that HA*-*Sir2_1−255_-Hst1_201−503_p can associate with both the Sir and Sum1 complexes and function in an Hst1p- and Sir2p-like manner or interact with neither complex, and HA*-*Sir2_1−255_-Hst1_201−503_p would be nonfunctional in both Sir- and Sum1-mediated repression.

To determine whether HA*-*Sir2_1−255_-Hst1_201−503_p has Hst1p-like function, the ability of this chimera to repress p*_PES4_-HIS3* was examined. HA*-*Sir2_1−255_-Hst1_201−503_p completely suppressed an *hst1*Δ mutation (Figure 6B). In fact, the chimeric protein was more effective than Sir2p at repressing p*_PES4_-HIS3* (Figure 6B compared to Figure 4B). However, Sir2p is still present in these cells and could affect the results. To directly compare the abilities of HA*-*Sir2_1−255_-Hst1_201−503_p, Hst1p-HA, and HA-Sir2p to function in an Hst1p-like manner, each of these proteins was expressed in an *hst1*Δ *sir2*Δ strain, and *DTR1* expression was assayed by quantitative RT-PCR. The chimera was better at repressing *DTR1* expression than Sir2p and was equally able to repress *DTR1* expression as wild-type Hst1p (Figure 6C).

To determine whether this repression mediated by HA*-*Sir2_1−255_-Hst1_201−503_p resulted from a strong interaction with the Sum1 complex, co-immunoprecipitation studies were performed. When Sum1p was immunoprecipitated, HA*-*Sir2_1−255_-Hst1_201−503_p coprecipitated to an extent comparable to, if not greater than, wild-type Hst1p and was much greater than Sir2p (Figure 6D). From these results we propose that unique features in the C terminus of Hst1p specify an interaction with Sum1p.

To determine whether HA*-*Sir2_1−255_-Hst1_201−503_p has Sir2p-like function, the ability to silence the mating-type loci was examined by mating assays. HA*-*Sir2_1−255_-Hst1_201−503_p enabled the cells to mate in the absence of Sir2p to a level comparable to that seen with wild-type Sir2p (Figure 7A). The extent of mating was greater with HA*-*Sir2_1−255_-Hst1_201−503_p than in cells expressing only wild-type Hst1p (Figure 7A). To test if the mating ability of HA*-*Sir2_1−255_-Hst1_201−503_p resulted from an association with the Sir complex, we co-immunoprecipitated Sir4p with HA-Sir2p, Hst1p-HA, and HA*-*Sir2_1−255_-Hst1_201−503_p. Results from these experiments showed an interaction between HA*-*Sir2_1−255_-Hst1_201−503_p and Sir4p comparable to that of wild-type Sir2p (Figure 7B). We conclude that there is a critical component in the N terminus of Sir2p that specifies Sir2p to interact with the Sir complex. The ability of the chimeric Sir2–Hst1 protein to suppress both *hst1*Δ and *sir2*Δ mutations suggests that different regions of the protein are involved in conferring specificity for the Sir and Sum1 complexes.

**Figure 7:**
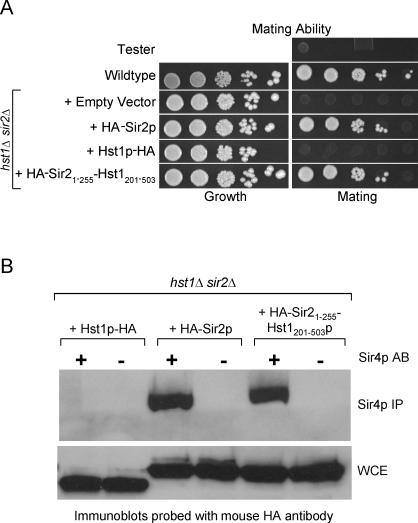
The Sir2_1−255_-Hst1_201−503_p Chimera Functions like Sir2p (A) The ability of Sir2p, Hst1p, and HA-Sir2_1−255_-Hst1_201−503_p to silence *HML* was assessed by a mating assay. An *hst1*Δ *sir2*Δ (LRY333) strain was transformed with an empty vector (pRS416), *HA*-*SIR2* (pRO298), *HST1*-HA (pLR30), and HA-Sir2_1−255_-Hst1_201−503_ (pLR488), and a ten-fold dilution series of each strain was mated against a MATα tester strain (LRY1022). Prototrophic diploids were selected on minimal plates. A 10-fold dilution series of the tester strain was plated on minimal plates as a negative control. (B) The association of HA-Sir2_1−255_-Hst1_201−503_p with Sir4p was assessed by co-immunoprecipitation. Sir4p was immunoprecipitated from whole-cell extracts from the strains used in (A), and the precipitated material was examined by immunoblotting with an α-mouse HA antibody to detect HA-Sir2p, Hst1p-HA, or HA-Sir2_1−255_-Hst1_201−503_p.

A recent study [42] also analyzed Sir2p and Hst1p interaction domains by using chimeric molecules and obtained similar results. This study determined that amino acids 12–209 in the N terminus of Sir2p were important for recruiting the protein to the Sir complex, consistent with our chimera analysis. Additionally, it was shown that two nonconserved amino acids in the catalytic core of Hst1p, Q324, and I325, were critical for recruitment to the Sum1 complex. Together, these results strongly indicate the presence of two different domains in Sir2p and Hst1p that confer substrate specificity for the Sir or Sum1 complex.

### The Nonduplicated Kl*SIR2* Functions in *SIR2*- and *HST1*-Like Repression

As outlined in the introduction, *SIR2* and *HST1* arose by gene duplication, and it is possible that the ancestral deacetylase interacted with both the Sum1 and Sir complexes. To test this model, we examined the function of the single *SIR2/HST1* gene from K. lactis, a species known to have diverged from S. cerevisiae before the whole genome duplication [25,26]. If the ancestral *SIR2/HST1* gene possessed only the function of *ScSIR2* or *ScHST1* and the other function evolved after the duplication, the gene having the new function would be expected to have experienced accelerated evolution compared with the gene retaining the original function. However, there appears to have been no accelerated evolution of either ScSir2p or ScHst1 compared to KlSir2p (Figure 8A) [26], an observation more consistent with a partitioning of functions after the duplication.

**Figure 8:**
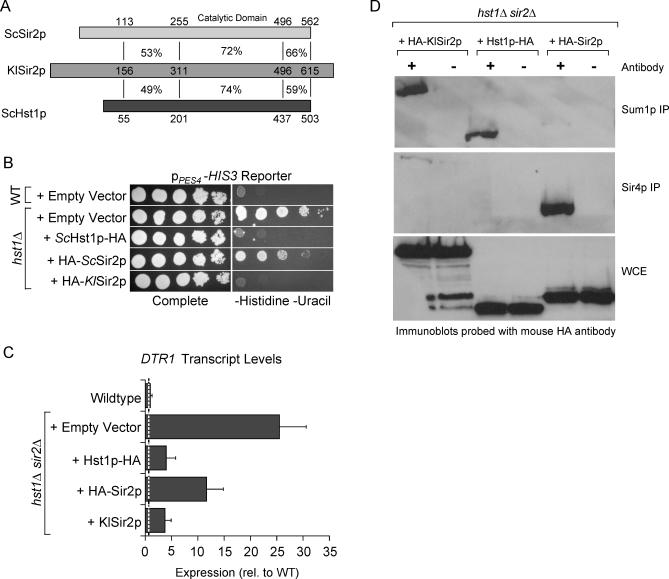
Hst1p Function Is Conserved in KlSir2p (A) The pairwise sequence identities between domains of KlSir2p, ScSir2p, and ScHst1p are shown. FASTA alignment software was used to calculate percent identity (and percent similarity) for each pairwise comparison. (B) The p*_PES4_-HIS3* reporter was used to assay repression by HA-KlSir2p (pLR490) and Hst1p-HA (pLR30) in an *hst1*Δ strain (LRY1545). A ten-fold dilution series of each strain was plated on rich medium and medium lacking histidine and uracil (to maintain plasmids) and photographed after 3 d growth at 30 °C. (C) *DTR1* gene expression of *hst1*Δ *sir2*Δ (LRY333) cells transformed with HA-Kl*SIR2* (pLR490) were compared to wild-type (W303-1A) expression. Data for the empty vector, Hst1p-HA and HA-Sir2p, are the same as shown in Figure 6C. (D) Association of HA-KlSir2p with Sir4p and Sum1p. Sir4p and Sum1p were immunoprecipitated from whole-cell extracts from strains used in (C), and the precipitated material was examined by immunoblotting with an α-mouse HA antibody to detect HA-Sir2p, Hst1p-HA, or HA-KlSir2p. The Hst1p–Sum1p and Sir2p-Sir4p co-immunoprecipitation samples are the same as those shown in Figures 6D and 7B, respectively.

The initial identification of Kl*SIR2/HST1* (referred to hereafter as Kl*SIR2*) reported that overexpression of Kl*SIR2* in S. cerevisiae was able to partially suppress a *sir2*Δ mating defect [43]. We did not observe suppression of a *sir2*Δ mating defect by KlSir2p (unpublished data); however, this could be due to differences in expression between our work and previously reported findings. Nevertheless, we could detect a weak interaction between KlSir2p and ScSir4p in co-immunoprecipitation experiments (Figure 8D). Subsequent studies of Kl*SIR2* revealed a role in silencing the mating-type loci in K. lactis [44]. Therefore, it has clearly been demonstrated that Kl*SIR2* has *SIR2*-like function both in K. lactis as well as in S. cerevisiae.

To test whether Kl*SIR2* is able to function in Hst1p-mediated repression in *S. cerevisiae,* Kl*SIR2* was cloned into a low-copy plasmid such that Kl*SIR2* has an N-terminal HA epitope tag and is expressed from the Sc*SIR2* promoter. This plasmid was used to transform the *hst1*Δ p*_PES4_-HIS3* strain of S. cerevisiae. Immunoblot analysis showed that Kl*SIR2* was stably expressed in S. cerevisiae (Figure 8D). There was complete repression of the p*_PES4_-HIS3* reporter by HA-Kl*SIR2,* with no observed difference from the wild-type level of repression (Figure 8B). We also examined the ability of KlSir2p to repress midsporulation genes in an *hst1*Δ *sir2*Δ background by analyzing *DTR1* expression levels and found that KlSir2p repressed *DTR1* to a level comparable to ScHst1p and better than ScSir2p (Figure 8C).

To test if this repression was a result of an interaction with the S. cerevisiae Sum1 complex, we immunoprecipitated ScSum1p and found that HA-KlSir2p coprecipitated (Figure 8D). From this data, as well as studies described previously [43,44], we conclude that the preduplicated Kl*SIR2* carries out both *SIR2-* and *HST1-*like functions. These data provide further evidence that the ancestral *SIR2/HST1* had dual functions that diverged after duplication.

## Discussion

In this study we provide evidence that in *S. cerevisiae,* the deacetylase Sir2p substitutes for Hst1p in its absence. Deletion of both *HST1* and *SIR2* results in dramatically greater derepression of Sum1p-repressed genes than observed in an *hst1*Δ background. This additional derepression is not observed when other related NAD^+^-dependent deacetylases or other silencing factors are deleted (Figure 1). Furthermore, Sir2p localizes to the promoters of Hst1p-repressed loci through an interaction with the Sum1 complex (Figure 2) and acts as a histone deacetylase at these loci in the absence of Hst1p (Figure 3). Based on these results, we propose a substitution model rather than genetic redundancy to explain the overlapping roles of this duplicate gene pair. We consider this phenomenon as substitution and not redundancy because Sir2p-mediated repression at Hst1-regulated genes is not as proficient as wild-type Hst1p-mediated repression and only occurs when Hst1p is absent. We propose that this substitution by Sir2p in an *hst1*Δ background accounts for some of the difference in derepression observed between *hst1*Δ and *sum1*Δ strains, although Sir2p substitution did not account for the entire difference in derepression phenotypes between *hst1*Δ and *sum1*Δ strains. It is possible that another deacetylase may also have limited ability to substitute, or it could be that Sum1p has repressive properties that are independent of a deacetylase.

It has also been observed by others that Hst1p substitutes for Sir2p in a *sir2*Δ background. Overexpression of Hst1p from a high copy plasmid partially suppresses *sir2*Δ mating defects in MATα cells [17]. However, this suppression does not completely restore function, as genetic redundancy would predict, because mating efficiency is still about 30-fold lower than in a wild-type strain. Given that overexpression of Hst1p is required to observe this effect, Hst1p may be less capable of substituting for Sir2p than Sir2p is capable of substituting for Hst1p. The requirement for multiple Sir2p–Sir4p complexes to silence a single *HMR* locus may also reduce the ability of Hst1p to substitute for Sir2p. This is in contrast to what is considered to be a single Hst1p–Sum1p complex required for repression, which would make it easier for Sir2p to substitute for Hst1p, even if the affinity of Hst1p for the Sir complex were comparable to the affinity of Sir2p for the Sum1 complex. Regardless, this previously published result is consistent with our model of the duplicated *SIR2*–*HST1* gene pair acting as substitutes for each other. This type of biological phenomenon has been proposed previously as the imposter model, with some controversy, for the MAP kinases Fus3p and Kss1p in S. cerevisiae [45,46]. However, our study has further developed this model to consider the evolutionary relationships between substituting proteins.

This substitution is likely a consequence of *SIR2* and *HST1* originating by duplication. Duplication has been proposed to be a strong evolutionary force because it generates a source of new genetic material that is free of selective constraint [1]. Duplicated genes have two ultimate fates: degeneration or preservation in the genome. Clearly *SIR2* and *HST1* have been retained. Two models have been proposed outlining the steps towards preservation. The classical model proposed that the only way to preserve duplicated genes is through neofunctionalization, in which one of the duplicate genes evolves a new function by acquiring beneficial mutations, while the other gene retains the original function. In such a case, it is predicted that the gene with the new function will experience a more rapid change in sequence, i.e., “accelerated evolution,” compared with the duplicate retaining the original function. A more recent paradigm for the preservation of duplicated genes has been proposed [47,48] to account for the much larger retention of duplicate genes than the classical model would predict. This new model of duplication, degeneration, and complementation (DDC) states that if the ancestral gene had multiple functions, duplicate genes can each lose one of the original functions by degenerative mutations, while still retaining a different ancestral function. The DDC mechanism was originally proposed in the context of *cis*-regulatory elements of duplicated gene pairs. However, our work suggests that the DDC mechanism can also act on protein coding sequences.

This study provides evidence to suggest that the ancestral *SIR2–HST1* gene provided both *SIR2*- and *HST1*-like functions. After the duplication, *SIR2* and *HST1* subfunctionalized to evolve into distinct *SIR2* and *HST1* genes with non-overlapping functions. By using K. lactis as a representative nonduplicated species, we found that the single *HST1*/*SIR2* gene completely suppressed an *hst1*Δ mutation in S. cerevisiae (Figure 8). Previous studies have reported that Kl*SIR2* contributes to silencing the *HM* loci in K. lactis [44] and can partially suppresses a *sir2*Δ mating defect in S. cerevisiae [43]. We have extended this analysis to show that KlSir2p can interact with Sir4p in S. cerevisiae (Figure 8). Together, these results indicate that the preduplicated *HST1/SIR2* is likely to have had both functions. It is probable that the Sir2 family has diversified by this type of mechanism.

Results from our functional characterization of the chimeric Sir2_1−255_-Hst1_201−562_ as well as those reported elsewhere [42] provide preliminary evidence that the evolution of *SIR2* and *HST1* may have followed a DDC mechanism. Two different specificity determinants in Sir2p and Hst1p have been found; a domain specific for determining an interaction with the Sum1 complex residing in the C terminus of Hst1p (Figure 6), specifically Q324 and I325 [42], and a domain specific for conferring an interaction with the Sir complex in amino acids 12–209 of the N terminus of Sir2p (Figure 7) [42]. These interaction domains have likely been conserved subsequent to the duplication. We propose a scenario in which, after the duplication, Hst1p acquired degenerative mutations in the N-terminal domain that interacts with the Sir complex, leading to the loss of affinity for Sir4p, yet maintaining its ability to interact with the Sum1 complex. Sir2p, on the other hand, acquired degenerative mutations in the C-terminal domain required for interaction with the Sum1 complex, leading to reduced affinity for the Sum1 complex, while maintaining a strong interaction with the Sir complex. Nevertheless, Sir2p has retained an interaction domain for the Sum1 complex, although it has a weaker affinity for this complex than Hst1p (Figure 2). Interestingly, of the two amino acids Q324 and I325, important for Hst1p specificity for the Sum1 complex in *S. cerevisiae,* only the isoleucine is conserved in K. lactis (K434 and I435). However, KlSir2p can fully suppress an *hst1*Δ mutation in S. cerevisiae (Figure 8). There may be additional residues in Hst1p that confer an interaction with the Sum1 complex but are conserved between ScHst1p, ScSir2p, and KlSir2p.

In this study we suggest that the particular evolutionary path taken as duplicated genes diverge from one another may be an important indicator of their potential contribution to genetic robustness. Duplicates that have subfunctionalized through a DDC mechanism may be more likely to substitute for each other than duplicates that display accelerated evolution or neofunctionalizion. *SIR3* and *ORC1* represent a pair of duplicated genes arising from the whole genome duplication that, in contrast to *SIR2* and *HST1*, experienced accelerated evolution [26]. Orc1p is an essential component of the origin recognition complex. Deletion of *ORC1* results in lethality, and Sir3p cannot complement an *orc1* mutation. Likewise, Orc1p cannot suppress a *sir3*Δ mating defect [49]. *ORC1* and *SIR3* are clearly an example of a duplicated gene pair that does not provide genetic robustness.

These results illustrate how gene duplication can provide genetic robustness against null mutations. It has been shown in S. cerevisiae that genes with duplicates are significantly more likely to have a weaker fitness defect phenotype compared to nonduplicated genes [6,50]. Here, we present data revealing that duplication provides genetic robustness through substitution not redundancy. This is an important distinction because about 550 duplicated gene pairs in S. cerevisiae were retained after the genome duplication [25,26], and many of these duplicates have diverged from each other [26]. It is quite likely that there are other duplicate genes, in addition to *SIR2* and *HST1,* which in wild-type backgrounds have nonoverlapping functions, yet, are able to substitute for one another in the event of a deletion. The biological significance of this phenomenon will be reflected in a null phenotype that underestimates or masks the real function of the deleted gene. Thus, one should apply caution in interpreting deletion phenotypes, particularly if it is known that the gene of interest has a retained duplicate. Our study also demonstrates that, in the case of an enzyme, the use of an inactivating mutation that abolishes enzymatic activity may be more useful in characterizing protein function than a complete deletion because such inactivating mutations retain the protein in the cell and thereby prevent an alternative protein from taking its place.

Finally, we can draw some conclusions about the relationship between different transcriptional repression mechanisms. It is clear from this study that deacetylation is an important component of Sum1p-mediated repression, as it is in Sir-mediated silencing. However, there is no intrinsic property of the deacetylase that determines whether it will act in a promoter-specific or regional manner (Figure 6). The results described here are consistent with our previous results indicating that a mutant form of Sum1p does spread and that this spreading requires the deacetylase activity of Hst1p rather than Sir2p [36]. Therefore, the tendency for a repressor complex to spread or not to spread is probably a function of the DNA or histone binding proteins with which the histone deacetylase associates. Sir2p is able to spread because its partners, Sir3p and Sir4p are able to spread. In fact, Sir3p and Sir4p can spread in the absence of Sir2p deacetylase activity when the histone tails mimic a deacetylated state [51], supporting the model that the role of Sir2p is to provide a substrate for its partners to bind. In contrast to Sir2p, Hst1p does not spread because its partner, Sum1p, normally does not spread [36]. This model is consistent with the hypothesis that the single ancestral histone deacetylase associated with both spreading (Sir) and nonspreading (Sum1) complexes.

## Materials and Methods

### Yeast strains.

Strains used in this study were all derived from W303-1a (Table 1). The *hst1*Δ*::KanMX*, *HST1*-HA, myc*-SUM1* [39], and *hst1-N291A* [36] alleles were described previously. The *sir3*Δ*::LEU2* and *sir2*Δ*::URA3* alleles were obtained from J. Rine (unpublished data). The *sir2*Δ*::TRP1, hst2*Δ*::TRP1, hst3*Δ*::TRP1* and *hst4*Δ*::TRP1* alleles were complete deletions of the open reading frames generated by one-step gene replacement. To generate the p*_GAS2_*-*HIS3* and p*_PES4_*-*HIS3* reporter alleles, the open reading frames of *GAS2* and *PES4* were replaced precisely with the *HIS3* open reading frame. The correct integration was confirmed by PCR using primers flanking the sites of recombination. These alleles were moved into various genetic backgrounds (as described in Table 1) through standard genetic crosses.

**Table 1:**
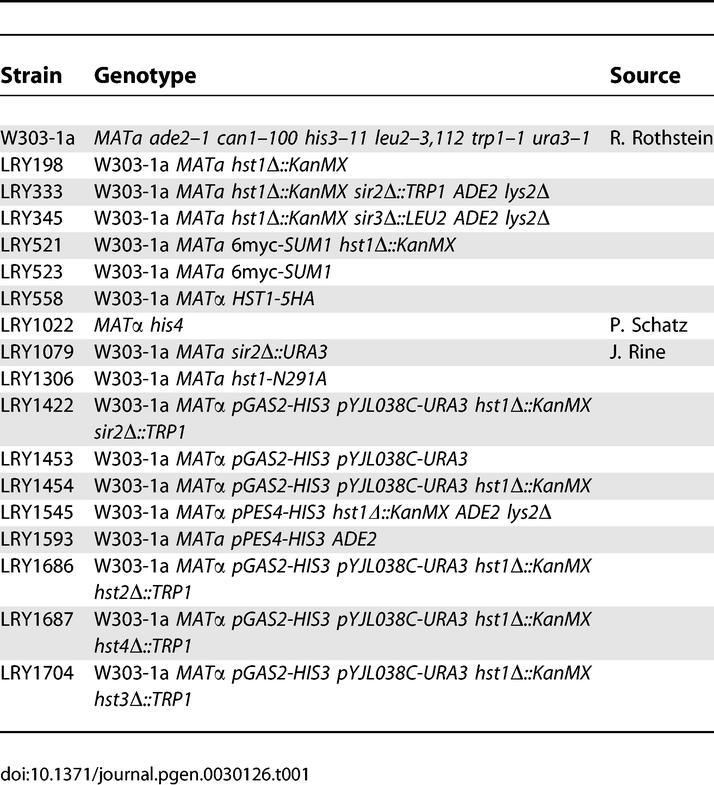
Strains Used in This Study

### Plasmids.

Plasmids used in this study are described in Table 2. The plasmid containing *HST1*-HA (pLR30) has been previously described [39]. To generate plasmid pLR488 expressing the chimeric *SIR2*–*HST1* protein, the N terminus of *SIR2* (amino acids 1–255) was amplified from genomic DNA, with the 5′ primer containing the recognition site for EcoRI and the 3′ primer containing 20 base pairs of overlapping homology to the start of the catalytic core of *HST1*. The C terminus of *HST1* (amino acids 201–503) was amplified from genomic DNA, with the 5′ primer containing 20 base pairs of overlapping homology to the *SIR2* sequence just upstream of the catalytic core sequence and the 3′ primer containing the recognition sequence for AgeI restriction endonuclease. A second PCR reaction was performed in which equimolar amounts of the *SIR2* N terminus amplicon and the *HST1* C terminus amplicon were pooled in a 25-μl PCR reaction and allowed to run in the thermocycler for five cycles, after which an additional 25 μl of reaction mix containing the 5′ oligonucleotide used previously for the *SIR2* amplification and the 3′ oligonucleotide used previously for the *HST1* amplification were added to the initial PCR reaction and allowed to cycle for 25 more rounds. The PCR product was cloned into the EcoRI and AgeI sites of pRO298, thereby replacing the *SIR2* open reading frame with the chimeric *SIR2–HST1* gene while retaining the N-terminal HA tag. The correct plasmid was verified by restriction enzyme analysis and sequencing. Expression of the HA-Sir2_1−255_-Hst1_201−503_ chimeric molecule was confirmed by immunoblotting. To generate plasmid pLR490 containing Kl*SIR2,* Kl*SIR2* was amplified from genomic DNA from a wild-type K. lactis strain (SAY45, from S. Astrom). The 5′ primer contained an MfeI site, and the 3′ primer contained an AgeI site. The resulting PCR product was cloned into the EcoRI and AgeI sites of pRO298, thereby replacing the Sc*SIR2* sequence with Kl*SIR2*. The correct plasmid was verified by restriction enzyme analysis and sequencing. Expression of HA-KlSir2p in S. cerevisiae was confirmed by immunoblotting.

**Table 2:**
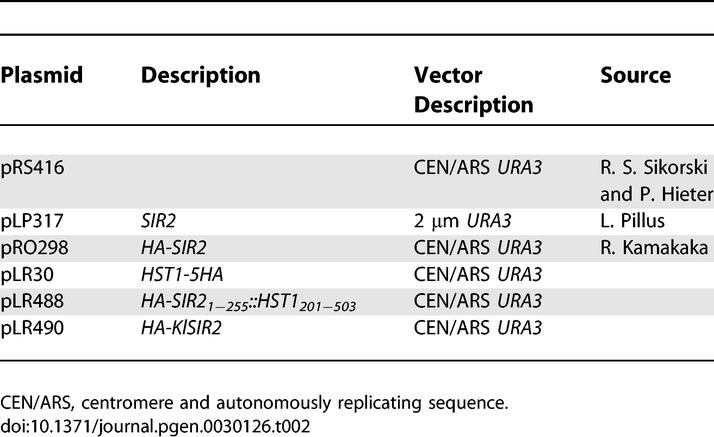
Plasmids Used in This Study

### Gene expression analysis.

RNA was isolated from two independent logarithmically growing cultures of each strain as previously described [52]. To remove DNA, 3 μg of RNA was treated with 3 units DNAseI (Promega, http://www.promega.com) and 1× DNase Buffer (Promega) in a final volume of 30 μl and incubated for 30 min at 37 °C. The DNase was inactivated by the addition of 3 μl of STOP solution (Promega) and incubation at 65 °C for 15 min. To verify that there was no contaminating DNA, 1 μl of DNAse-treated material was used in a PCR reaction containing primers to amplify the *ACT1* transcript. A lack of product indicated successful removal of DNA. We used 1 μg of DNA-free RNA for cDNA synthesis by addition of 1 μl 10 mM dNTPs and 1 μl oligo dT_16_ (500 ng/μl) and incubation at 65 °C for 5 min, followed by a quick chill on ice. A master mix of 4 μl 5× first strand buffer (Invitrogen, http://www.invitrogen.com), 2 μl 0.1 M DTT, and 1 μl RNAseOUT (Invitrogen) was added to the samples. The resulting reaction was incubated at 37 °C for 2 min at which point 1 μl of M-MLV-RT (Invitrogen) is added to the reaction and incubated for 50 min at 37 °C followed by a 15-min incubation at 70 °C to inactivate the enzyme. We subsequently analyzed one-fortieth of the resulting cDNA by real time-PCR in the presence of SYBR Green on a Bio-Rad iCycler (http://www.bio-rad.com) to quantify the relative amounts of mRNA transcripts. Duplicate qPCR reactions were performed to ensure consistency. The standard curve was generated with genomic DNA isolated from the wild-type strain (W303-1a). Oligonucleotide sequences are provided in Table 3. Data were analyzed with iCycler iQ Optical System Software (Bio-Rad). *DTR1* and *SPS1* transcript levels were normalized to *ACT1* transcript levels. To determine fold-induction, *DTR1* and *SPS1* transcript levels were normalized to the wild-type strain. Results reflect the average fold induction (relative to a wild-type strain) of two independent cultures for each strain background, each analyzed in duplicate qPCR reactions. The standard deviation was calculated from the difference in fold induction of the two independent cultures from the mean.

**Table 3:**
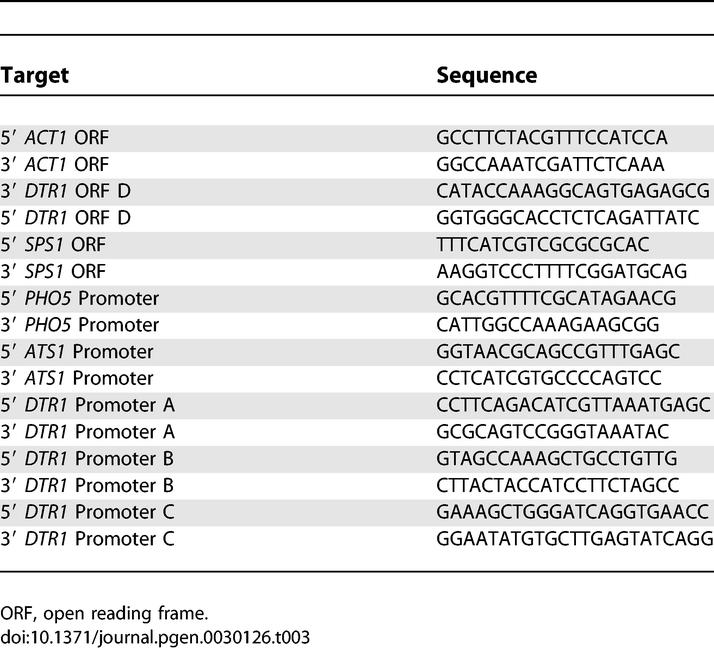
Oligonucleotides Used in This Study

### Chromatin immunoprecipitation.

ChIPs were performed as previously described [39] using ten optical density equivalents of cells and 2–4 μl anti-HA tag antibody (Upstate Biotechnology 05–902, http://www.upstate.com), 2 μl of antibodies against acetyl-lysine 8 or acetyl-lysine 16 of histone H4 (Upstate Biotechnology 07–328 and 06–762), or 3 μl anti-myc tag antibody (Upstate Biotechnology 06–549). For immunoprecipitation of HA-Sir2p and Hst1p-HA, a second crosslinking agent was used [53]. A total of 50 optical density units of cells were harvested by centrifuging at 2,700 rpm for 5 min and resuspended in 1× ice cold DMA (10 mM dimethyl adipimidate, 0.1% DMSO, and 1× PBS) and crosslinked for 45–60 min at room temperature. After crosslinking with DMA, cells were washed twice with cold 1× PBS, resuspended in 50 ml 1× PBS, and treated with 1% formaldehyde for 45–60 min at room temperature. The DNA was sheared by sonication to an average size between 500 to 1,000 bp in all experiments. ChIP samples were analyzed by qPCR using a standard curve prepared from input DNA. The amounts of the immunoprecipitated DNA at the experimental promoter *(DTR1)* and a control promoter *(ATS1)* were determined relative to the input DNA, and then the enrichment of the *DTR1* promoter was determined relative to the control locus *ATS1*. Enrichment is considered significant if the ratio of experimental to control region equals two or higher. Oligonucleotide sequences are provided in Table 3. Results reflect the relative immunoprecipitation of two independent cultures for each strain background, and the standard deviation was calculated from the difference in fold induction of the two independent cultures from the mean. To determine the relative acetylation level of Lys8 and Lys16 of H4 (Figure 3) in various strain backgrounds, normalized *DTR1* IP levels were quantified relative to the wild-type strain. To determine differences in nucleosome occupancy in the various strain backgrounds, an independent ChIP using an antibody against the H4 core domain (Upstate Biotechnology 07–108) was performed. Results in Figure 3 depict relative acetylation levels of Lys8 and Lys 16 of H4 for each strain that accounts for strain differences in nucleosome occupancy.

### Co-immunoprecipitations.

Co-immunoprecipitations were performed as previously described [39] using 30 optical density equivalents of cells grown in media lacking uracil to ensure plasmid retention. The whole-cell lysates were incubated for 4 h at 4 °C with 5 μl of antibody. For Sum1p immunoprecipitations, serum from a guinea pig inoculated with a C-terminal fragment of Sum1p was used (Pocono Rabbit Farm, http://www.prfal.com). For Sir4p immunoprecipitations, serum from a rabbit inoculated with Sir4p was used [54]. After incubation with the antibody, 60 μl of protein A agarose beads (50% slurry from Upstate) were added and samples were rotated at 4 °C overnight. Samples and whole-cell extracts were electrophoretically fractionated on 7.5% polyacrylamide–SDS gels, transferred to nitro-cellulose membranes (Amersham, http://www.amersham.com), and probed with mouse polyclonal α-HA antibody (Upstate Biotech 05–904).

### Immunoblotting.

Whole-cell protein samples were prepared from three optical density equivalents of cells grown in medium lacking uracil to ensure plasmid retention. Trichloroacetic acid (TCA) was added to the culture medium to a final concentration of 10%, and the cells were incubated on ice for 20 min. Cells were pelleted, washed with Tris pH 8.0, and resuspended in 75 μl 3× protein sample buffer. Cells were lysed by vortexing in the presence of glass beads and subsequently incubated at 95 °C. Whole-cell protein extracts were electrophoretically fractionated on 7.5% polyacrylamide-SDS gels, transferred to nitro-cellulose membranes (Amersham), and probed with rabbit α-Sir2p (from J. Rine), rabbit α-HA antibody (Upstate Biotech 05–902), or 3-phosphoglycerate kinase (Molecular Probes/Invitrogen A-6457).

### Mating assays.

One optical density equivalent of cells was collected from logarithmically growing cultures by centrifugation and resuspended in 100 μl YM (yeast minimal) medium. For each strain, ten-fold serial dilutions were prepared, and 3 μl of each sample in the dilution series was spotted onto a YPD plate to monitor growth. To assay mating, an equal volume of the tester strain LRY1022 (MATα *his4*) at 10 OD equivalents /ml in YPD was mixed with each sample in the dilution series, and 3 μl of this mixture was spotted onto YM plates to select for the growth of prototrophic diploids. Yeast were grown at 30 °C for 3–4 d and subsequently photographed.

### Reporter assays.

One optical density equivalent of logarithmically growing cells was collected by centrifugation in a microcentrifuge and subsequently resuspended in 100 μl YM medium. For each strain, ten-fold or five-fold serial dilutions were prepared, and 3 μl of each sample in the dilution series was spotted onto complete medium to monitor overall growth and medium lacking histidine or lacking histidine and uracil to monitor Hst1p-mediated repression. Uracil was omitted to maintain the plasmids in the p*_PES4_-HIS3* reporter assays. Yeast were grown at 30 °C for 3–4 d and subsequently photographed.

## Abbreviations

ChIP: chromatin immunoprecipitation
DDC: duplication, degeneration, and complementation
H4: histone H4
K8: lysine 8
K16: lysine 16
MSE: middle sporulation element
